# Closed‐loop automated oxygen control in late preterm and term, ventilated infants: A randomised controlled trial

**DOI:** 10.1111/apa.17549

**Published:** 2024-12-14

**Authors:** Ourania Kaltsogianni, Allan Jenkinson, Christopher Harris, Eleanor Jeffreys, Oishi Sikdar, Anne Greenough, Theodore Dassios

**Affiliations:** ^1^ Women and Children's Health, School of Life Course Sciences, Faculty of Life Sciences and Medicine King's College London London UK; ^2^ Neonatal Intensive Care Centre King's College Hospital NHS Foundation Trust London UK

**Keywords:** closed‐loop automated oxygen delivery, hyperoxemia, term infant

## Abstract

**Aim:**

To compare the time spent above the target oxygen saturation range (SpO_2_ > 96%) and the duration of supplemental oxygen between ventilated infants receiving closed‐loop automated oxygen control (CLAC) or manual oxygen control in late preterm and term ventilated infants.

**Methods:**

Infants were randomised to receive CLAC or manual oxygen control from recruitment and within 24 h of mechanical ventilation until successful extubation.

**Results:**

Forty infants with a median (IQR) gestational age of 37.4 (35.9–38.5) weeks were studied at a corrected postmenstrual age of 37.6 (36.0–38.7) weeks. In infants randomised to CLAC (*n* = 18) the time spent above the target oxygen saturation range was reduced by 20% (*p* < 0.001), and the time spent in the target range (92%–96%) was increased by 32% (*p* < 0.001) and the time spent in hyperoxia was reduced (*p* = 0.003). CLAC reduced the time spent in hypoxemia (SpO_2_ < 85%) (*p* = 0.017) and there were fewer manual adjustments to the inspired oxygen concentration (FiO_2_) (*p* < 0.001). There was no significant difference in the duration of supplemental oxygen (*p* = 0.271).

**Conclusion:**

CLAC in ventilated infants born at or near term was associated with reduced time spent in hyperoxemia, more time spent in the target oxygen range, and fewer manual adjustments to the FiO_2_.

**Abbreviations**
CDH: congenital diaphragmatic hernia
CLAC: closed‐loop automated oxygen control
CNS: central nervous system
FiO_2_
: inspired oxygen concentration
HIE: hypoxic‐ischemic encephalopathy
ICE: infant cooling evaluation
ICU: intensive care unit
IQR: interquartile range
MAS: meconium aspiration syndrome
PID: proportional integral derivative
PPHN: persistent pulmonary hypertension of the newborn
RCT: randomised controlled trial
REC: Research Ethics Committee
SpO_2_
: peripheral oxygen saturation
TTN: transient tachypnoea of the newborn
TWA: time‐weighted average

**Key notes**

- There are few studies of closed‐loop automated oxygen control (CLAC) in term infants.
- In a randomised controlled trial, CLAC increased the time spent in the target oxygen range and reduced the time spent in hyperoxemia (SpO_2_ > 98%) and hypoxemia (SpO_2_ < 85%) in ventilated infants born ≥34 weeks of gestation, with fewer manual adjustments to the inspired oxygen concentration.
- Appropriately powered clinical trials are required to determine if CLAC influences long‐term outcomes.

## 1 INTRODUCTION

The most frequent cause for neonatal unit admission both in term and preterm neonates is the need for respiratory support.^1^ Term infants require respiratory support for a variety of reasons including meconium aspiration syndrome (MAS), respiratory distress, transient tachypnoea of the newborn (TTN), pneumonia, sepsis and pulmonary hypertension. Late preterm infants (those born between 34 and 36^+6^ weeks of gestation) account for the majority of preterm births and, due to the interruption to normal lung development, are more likely to need mechanical ventilation and at higher risk of respiratory morbidity when compared to term‐born neonates.

Neonates with respiratory distress frequently require supplemental oxygen, but both excessive or inadequate dosing can have detrimental effects.^2^, ^3^, ^4^ Compliance with the achievement of oxygen saturation targets can be poor (12). Neonatal patients experience frequent fluctuations in their oxygen saturation levels.^5^ Closed‐loop automated oxygen control (CLAC) systems increase the time spent within oxygen saturation targets with fewer manual adjustments to the inspired oxygen concentration (FiO_2_).

There are several commercially available devices for automated oxygen titration that use different algorithms. One of them (Oxygenie software [Inspiration Healthcare, South Croydon, UK]) has been shown to be effective in improving compliance with oxygen target achievement in preterm infants and preventing hypoxemia and hyperoxemia with fewer manual adjustments of the inspired oxygen.^6^ A meta‐analysis including 13 randomised controlled trials (RCTs) in preterm infants^7^ concluded that CLAC significantly reduced the percentage of time spent in hyperoxemia (SpO_2_ > 98%). In addition, previous studies demonstrated that CLAC could facilitate earlier weaning of the FiO_2_ when compared to manual oxygen control in preterm infants receiving invasive^8^ or non‐invasive respiratory support.^9^

To date, most studies have included very preterm or low birth weight infants. Yet, babies born at or near term are also vulnerable to the risks related to supplementary oxygen with hypoxemia and hyperoxemia causing significant respiratory morbidity.^10^, ^11^ In a crossover study, CLAC improved the achievement of oxygen saturation targets, reduced hyperoxemic episodes and facilitated weaning of the inspired oxygen concentration in ventilated infants born at or near term.^12^ The aim of this study was to determine, whether in a RCT, CLAC compared to manual oxygen control reduced the time spent in hyperoxemia and supplemental oxygen duration in ventilated infants born at or greater than 34 weeks of gestation.

## 2 PATIENTS AND METHODS

A non‐blinded, RCT was undertaken at a tertiary neonatal unit (King's College Hospital NHS Foundation Trust, London, UK) between December 2022 and June 2024. Ventilated infants born at or above 34 weeks of gestation and within 24 h of initiation of mechanical ventilation were eligible for the study unless they had congenital cyanotic heart disease or were receiving high‐frequency oscillatory ventilation. Infants with congenital diaphragmatic hernia (CDH) were studied post‐operatively. The study was approved by the West Midlands‐Edgbaston NHS Research Ethics Committee (REC). Written, informed parental consent was obtained before recruitment.

Participants were randomised to receive either closed‐loop automated (intervention group) or manual (control group) adjustment of the oxygen concentration from recruitment to successful extubation.^13^ Randomisation using an online randomisation generator was used to determine an infant's study arm. Infants who failed extubation and required reintubation within 48 h continued in their initial study arm. If an infant was successfully extubated the study was completed. The nurse‐to‐patient ratio was according to the unit protocol determined by the patient's acuity and was 1:2 for all patients during the study.

Infants were ventilated on pressure‐limited timed‐cycled or volume‐targeted ventilation using SLE6000 neonatal ventilators and ventilation settings were manually adjusted by the clinical team as per the unit's protocol. For the duration of the study, participants were connected to the standard bedside monitor (Philips IntelliVue MX750) and a SpO_2_ cable (SLE uSPO_2_ cable) which was connected to the ventilator for continuous recording of SpO_2_ and FiO_2_ levels (standard care). The Philips monitor uses the Nellcor Neonatal SpO_2_ sensor. The SLE cable uses a Masimo SET Neonatal Pulse Oximetry Adhesive Sensor. Both sensors were applied to all study participants for the duration of the study. There was usually a difference of 1%–2% in SpO_2_ levels displayed with the Masimo probe providing lower readings. The SLE SpO_2_ function would activate an alarm for a SpO_2_ below or above the target range. The clinical team would react to the bedside monitor that represented standard care but any alarms from the ventilator would also be noted. Analysis was performed on data obtained via the SLE SpO_2_ cable. Oxygen saturation monitoring could be either pre‐ or post‐ductal unless there was a difference between them, then pre‐ductal readings would be obtained. In addition to standard care, infants in the intervention group were connected to the Oxygenie Auto‐O_2_ closed‐loop oxygen saturation monitoring software (Inspiration Healthcare, South Croydon, UK) which uses a proportional–integral–derivative (PID) algorithm.^14^

Manual adjustments to the FiO_2_ were allowed at any point during the study including the infants receiving automated oxygen control if deemed appropriate by the clinical team. The Oxygenie system has an alarm set to be activated if there is an increase in FiO_2_ by 30% from the baseline oxygen requirement.

Data were downloaded directly from the ventilator and consisted of second‐by‐second recordings of ventilator settings and paired SpO_2_ and FiO_2_ results. Data collected were the proportion of the time spent in hypoxemia (SpO_2_ < 92% and SpO_2_ < 85%) and severe hypoxemia (SpO_2_ < 80%), the percentage of time spent within the target oxygen range (SpO_2_: 92%–96%), above the patient's target oxygen saturation range (SpO_2_ > 96%), the time spent in hyperoxemia (SpO_2_ > 98%) and the percentage of time spent with a FiO_2_ > 0.3. When infants were weaned to room air (FiO_2_: 0.21), a SpO_2_ above the target range was considered as being within the target range as the FiO_2_ could not be weaned any further. The unit's protocol for oxygen titration was to adjust the FiO_2_ as required to maintain the SpO_2_ within the target range. This approach was taken during the manual and automatic oxygen control periods.

Demographic and outcome data were collected from the clinical records. Basic epidemiologic parameters such as gestational age, birth weight, corrected gestational age, postnatal age and weight at study enrolment, use of antenatal steroids, mode of delivery, doses of surfactant administered, birth plurality and diagnoses at enrolment were recorded for each infant.

The primary outcome was the percentage of time spent above the target oxygen saturation range (SpO_2_ > 96%). The secondary outcomes were the supplementary oxygen duration, the percentage of time with an oxygen requirement exceeding 13%, the number of days on mechanical ventilation and the length of neonatal unit stay.

### 2.1 Sample size calculation

A retrospective cohort study of 7784 mechanically ventilated adult patients demonstrated that the time‐varying intensity of oxygen exposure, as measured by daily time‐weighted average (TWA) arterial oxygen tension (PaO_2_) or TWA FiO_2_ was significantly associated with increased 28‐day mortality. The impact of the high‐level FiO_2_ was persistent over time and could directly impact mortality, independently from the presence of hyperoxemia. Therefore, we based our power calculation on this parameter.^15^

In an interim analysis of data from a randomised crossover study on CLAC in ventilated infants born at or above 34 weeks of gestation undertaken in our unit, the use of a closed‐loop oxygen system was associated with a reduction in the mean FiO_2_ delivery during hyperoxemia from 29.6% (manual oxygen control period) to 24.7% (automated oxygen control period, *p* = 0.018), a difference of 4.9%. The standard deviation for the FiO_2_ requirement of the infants at the beginning of the automated oxygen control period was 4.7%.^16^ Randomisation of 40 infants allowed the detection of a difference in the mean FiO_2_ during hyperoxemia of 4.9% with a 90% power at the 5% significance level.^17^

### 2.2 Analysis

Data were compared between infants in the intervention group and the control group. The data were assessed for normality with the Kolmogorov‐Smirnoff test and found not to be normally distributed. Hence, differences were tested for statistical significance using the Mann–Whitney U‐test. Categorical data were assessed using the Chi‐squared test. The analysis was undertaken using IBM SPSS version 28.0.1.1 (SPSS Inc., Chicago, IL, USA). Patients' data were analysed according to their randomised assignment group. Imputation of missing data was not performed. Ventilation settings and oxygen requirements at the beginning of the study were compared between infants in the two groups to determine if there were differences in respiratory disease severities.

## 3 RESULTS

Informed consent was obtained from 43 parents. Three infants were excluded because parents withdrew consent (*n* = 1) or due to technical issues relating to the Oxygenie controller that delayed the start of the study and they no longer required mechanical ventilation (*n* = 2). All included infants (*n* = 40) completed the study. There were 18 infants in the intervention group and 22 in the control group. The 40 infants had a median gestational age of 37.4 (interquartile range [IQR] 35.9–38.5) weeks and median birth weight of 2782 (IQR 2402–3172) grams and were studied at a median corrected post‐menstrual age of 37.6 (IQR 36.0–38.7) weeks (Table 1). Fourteen infants were ventilated following surgery for congenital abnormalities of the gastrointestinal tract (including gastroschisis, oesophageal or small bowel atresia and antenatal malrotation). Another three infants were studied post‐repair of left‐sided CDH. The remaining subjects had diagnoses of respiratory distress (*n* = 11), transient tachypnoea of the newborn (*n* = 1), hypoxic‐ischemic encephalopathy (*n* = 1), persistent pulmonary hypertension of the newborn (PPHN) (*n* = 4), neonatal seizures (*n* = 2), pulmonary haemorrhage (*n* = 1), sacrococcygeal teratoma (*n* = 1), axillary lymphangioma (*n* = 1) and diaphragmatic eventration (*n* = 1).

**TABLE 1:** Demographic characteristics of the infants included in the study.

	Infants randomised to CLAC	Infants randomised to manual oxygen control	*p*‐value
Total number of infants	*N* = 18	*N* = 22	
Birth			
Male sex	12 (67)	13 (59)	0.114
Gestational age (weeks)	37.3 (34.3–41.3)	37.4 (34.1–40.1)	0.913
Birth weight (g)	2715 (1905–5050)	2787.5 (1890–4265)	0.744
Antenatal steroids			
Complete course	2 (11)	2 (9)	0.731
Incomplete course	1 (6)	2 (9)	
Unknown	0	2 (9)	
Surfactant	2 (17)	5 (23)	0.316
APGAR score at 1 min	8 (3–9)	9 (2–9)	0.859
APGAR score at 5 min	10 (5–10)	9 (1–10)	0.239
In NICU			
Day of life at study	2 (0–9)	2 (1–16)	0.528
Hours ventilated at study	9.5 (1–23)	8 (1–339.6)	0.957
Day of life at extubation	4 (2–16)	4 (1–16)	0.754
CGA at extubation	37.9 (35.1–41.4)	37.9 (34.7–43)	1
Extubation success	18 (100)	20 (100)	n/a
Discharge			
Survived to discharge	18 (100)	22 (100)	n/a
Post‐menstrual age at discharge (weeks)	41.3 (35.6–50.4)	39.9 (36.7–44.7)	0.314
Weight at discharge (g)	3425 (1840–5590)	2972.5 (2145–4180)	0.103

*Note*: Data are presented as: *N* (%) or medians (range). Mann‐Whitney U‐test/ Chi‐square tests.

Abbreviation: CLAC, closed‐loop automated oxygen control.

There were no adverse events related to the study. There were no significant differences in the ventilator settings of the two groups at the beginning of the study (Table 2).

**TABLE 2:** Ventilator settings of the infants at the beginning of the study periods.

Ventilator parameters at the beginning of the study	Infants randomised to CLAC	Infants randomised to manual oxygen control	*p*‐values
Mean airway pressure (cm H_2_O)	10.05 (6–16.4)	9.05 (6–16)	0.216
Positive inspiratory pressure (cm H_2_O)	25 (11.2–30)	25 (16.2–30)	0.38
Positive end expiratory pressure (cm H_2_O)	5 (4.9–6)	5 (5–6.6)	0.245
Inspiratory time (s)	0.4 (0.38–0.45)	0.4 (0.38–0.5)	0.67
Rate (breaths per minute)	40 (30–55)	35 (20–50)	0.178
Inspired oxygen concentration (%)	34 (21–61)	28 (21–60)	0.177

*Note*: Data are presented as median (range).

Abbreviation: CLAC, closed‐loop automated oxygen control.

The median percentage of time spent above the target oxygen saturation range (SpO_2_ > 96%) and in hyperoxemia (SpO_2_ > 98%) was significantly lower for infants receiving CLAC (*p* < 0.011 and *p* = 0.003 respectively), but the mean FiO_2_ delivery with oxygen saturation levels above the target range (median: 27.8% (range: 21%–54.8%) [intervention group] versus median: 29.8% (range: 23.7%–43.2%) [control group], *p* = 0.547) was not significantly different. There were no significant differences in the time with oxygen saturation levels below the target range (SpO_2_ < 92%) (*p* = 0.141; Table 3) between the two groups, but infants in the intervention group spent significantly less time in hypoxemia (SpO_2_ < 85%) (*p* = 0.017), and the median FiO_2_ delivery during hypoxemia was significantly higher (*p* = 0.011). The time spent in severe hypoxemia (SpO_2_ < 80%) did not differ significantly between the two groups (*p* = 0.072).

**TABLE 3:** Hypoxemia and hyperoxemia by monitoring type.

	Infants randomised to CLAC (*N* = 18)	Infants randomised to manual oxygen control (*N* = 22)	*p*‐value
% time below target range^a^	4.2 (1.2–17.9)	5.4 (0.4–10.3)	0.141
% time in target range^b^	91.1 (63.3–98.7)	58.8 (9.6–94.2)	<0.001
% time above target range^c^	2.8 (0–25.2)	20.8 (0–86.7)	<0.001
% time in hypoxemia^d^	0.06 (0.01–0.5)	0.26 (0–2.7)	0.017
% time in hyperoxemia^e^	0.06 (0–2.4)	2.1 (0–47.44)	0.003
% time in severe hypoxemia^f^	0.02 (0–0.28)	0.05 (0–1.09)	0.072
SpO_2_ below target range	90 (87–91)	89.5 (89–91)	0.605
FiO_2_ below target range	29 (23.5–85)	25 (21–45)	0.011
SpO_2_ in target range	96.5 (94–99)	96.9 (94–99)	0.24
FiO_2_ in target range	21 (21–43)	21 (21–45)	0.661
SpO_2_ above target range	97 (96–99)	98 (97–99)	0.115
FiO_2_ above target range	25 (21–50.5)	28 (0–45)	0.331

*Note*: Data are presented as the medians (range). Mann‐Whitney U‐test.

Abbreviation: CLAC, closed‐loop automated oxygen control.

^a^ SpO_2_ < 92%.

^b^ SpO_2_: 92%–96%.

^c^ SpO_2_ > 96%.

^d^ SpO_2_ < 85%.

^e^ SpO_2_ > 98%.

^f^ SpO_2_ < 80%.

Infants receiving CLAC spent a greater proportion of their time within the target SpO_2_ range (*p* < 0.001). The median FiO_2_ requirement to achieve peripheral oxygen saturations within the target range did not differ significantly between the two groups (*p* = 0.661).

Significantly fewer manual adjustments were made to the FiO_2_ during CLAC (median: 0 [range: 0–1]) when compared with manual oxygen control (median: 10.5 [range: 1–37], *p* < 0.001).

There were no significant differences between the two groups in the number of days receiving supplemental oxygen (*p* = 0.271) (Table 4), the time spent with an oxygen requirement exceeding 30% (*p* = 0.586), the duration of mechanical ventilation (*p* = 0.699) and the length of neonatal unit stay (*p* = 0.321).

**TABLE 4:** Primary and secondary outcomes by monitoring type.

	Infants randomised to CLAC	Infants randomised to manual oxygen control	*p*‐value
% time above target range^a^	2.8 (0–25.2)	20.8 (0–86.7)	<0.001
Duration of supplementary oxygen treatment (days)	4.5 (1–24)	3 (1–18)	0.271
% time with an oxygen requirement >30%	8.5 (0–81.5)	15 (0.02–99.9)	0.586
Number of days on mechanical ventilation	3 (1–14)	3.5 (1–75)	0.699
Length of neonatal unit stay (days)	19 (4–106)	16.5 (5–39)	0.321

*Note:* Data are presented as the medians (range). Mann‐Whitney U‐test.

Abbreviation: CLAC, closed‐loop automated oxygen control.

^a^ SpO_2_ > 96%.

## 4 DISCUSSION

We have demonstrated in a RCT that automated oxygen control was associated with an increased percentage of time in the target oxygen saturation range, reduced time in hyperoxemia and hypoxemia and fewer manual adjustments to the inspired oxygen concentration.

In infants randomised to CLAC the time spent in the target oxygen range (92%–96%) was increased by 32.2% (*p* < 0.001). Those results confirm our previous findings from a randomised controlled crossover study of CLAC in late preterm and term‐born infants and are in agreement with data from preterm populations.^7^, ^18^, ^19^

The time spent below the target SpO_2_ range did not differ significantly between the two groups, but the time spent in hypoxemia (SpO_2_ < 85%) was significantly shorter in the CLAC arm. Results from previous studies have suggested automated oxygen control has not always been advantageous with regard to the time spent below the targeted SpO_2_ range. In some studies,^20^, ^21^, ^22^, ^23^, ^24^ automated oxygen control reduced the percentage of time spent below the targeted SpO_2_ range, but in others, it was less effective than manual control.^8^, ^25^, ^26^, ^27^, ^28^ In agreement with our findings, a systematic review^18^ demonstrated no significant difference in the percentage of time spent below the targeted SpO_2_ range between automated and manual oxygen control. Automated oxygen control though significantly reduced the percentage of time spent in severe hypoxemia (SpO_2_ < 80%) and the number of hypoxemic episodes.^18^ We have not demonstrated any effect of CLAC on the time spent in severe hypoxemia (SpO_2_ < 80%) but this percentage time was negligible in both study groups.

Closed‐loop automated oxygen control has been shown to reduce the percentage of time spent in hyperoxemia^7^, ^18^, ^19^, ^29^ and the number and duration of hyperoxemic episodes.^30^ A recent Cochrane review comparing automated versus manual oxygen control in preterm infants receiving invasive or non‐invasive respiratory support demonstrated that automated oxygen delivery reduced the time spent above the desired SpO_2_ range in those who were ventilated.^19^ In the subgroup receiving non‐invasive respiratory support, automated oxygen delivery was also effective in preventing hyperoxemia but the effect was smaller. In our cohort, the median oxygen requirement of the infants in both groups was below 30% during hypoxemia, hyperoxemia and when the SpO_2_ was within the target range, but still automated oxygen control led to an 18% reduction in the median time spent above the target oxygen saturation range. There were no significant differences in the time spent with an FiO_2_ > 0.3 probably due to the relatively low oxygen requirement and mild respiratory disease severity of our infants.

Hyperoxemia can lead to the generation of free radicals that induce membrane disruption and activate inflammatory pathways through lipid peroxidation, affecting multiple organ systems.^31^ Newborn infants have weak antioxidant defence mechanisms and exposure to hyperoxemia has been associated with lung, retina and central nervous system (CNS) in preterm infants.^32^ More mature infants are also vulnerable. In a RCT, term neonates resuscitated with 100% oxygen exhibited biochemical findings of oxidative stress even after 4 weeks of postnatal life.^33^ In addition, free radicals may promote pulmonary vasoconstriction and decrease the pulmonary vasodilator response to inhaled nitric oxide.^34^ In a secondary analysis of the data from the Infant Cooling Evaluation (ICE) trial, hyperoxemia within 2 h of birth significantly increased the risk of death or major disability at 2 years in infants with HIE.^35^ In a systematic review of 25 RCTs and 16 000 patients, liberal compared with conservative oxygen strategies increased in‐hospital and out‐of‐hospital mortality in acutely ill adults.^36^ Similarly, in a retrospective observational study involving more than 6000 paediatric ICU patients, severe hyperoxemia (PaO_2_ > 300 mmHg) was independently associated with in‐hospital mortality.^37^ Avoiding hyperoxemia in the neonatal period may improve outcomes.

Our study has strengths and some limitations. To our knowledge, this is the first RCT comparing closed‐loop automated oxygen control with manual oxygen control in late preterm and term‐born ventilated infants. Although many participants were ventilated for non‐respiratory disease we still demonstrated a significant improvement in the achievement of oxygen saturation targets. We have not demonstrated any significant differences in secondary outcomes but our study was not powered to detect such differences.

In conclusion, CLAC improved compliance with the achievement of oxygen saturation targets and reduced hyperoxemia in ventilated infants born at or near term with fewer manual adjustments to the inspired oxygen.

## AUTHOR CONTRIBUTIONS

**Ourania Kaltsogianni:** Writing – original draft; investigation; formal analysis. **Allan Jenkinson:** Investigation; formal analysis. **Christopher Harris:** Investigation; formal analysis. **Eleanor Jeffreys:** Investigation; formal analysis. **Oishi Sikdar:** Investigation; formal analysis. **Anne Greenough:** Conceptualization; writing – review and editing; supervision; project administration. **Theodore Dassios:** Conceptualization; writing – review and editing; supervision; project administration.

## FUNDING INFORMATION

This research was supported by the National Institute for Health Research (NIHR) Biomedical Research Centre at Guy's and St Thomas’ NHS Foundation Trust and King's College London. The views expressed are those of the authors and not necessarily those of the NHS, the NIHR or the Department of Health.

## CONFLICT OF INTEREST STATEMENT

The authors have no conflict of interest to declare.

## Supporting information


Data S1

## Data Availability

Data are available upon reasonable request.
