# Supplementary material for: Closed‐loop automated oxygen control in late preterm and term, ventilated infants: A randomised controlled trial
Source: Acta Paediatr. 2024 Dec 14;114(6):1222–8. doi: 10.1111/apa.17549 (PMC12066902; doi:10.1111/apa.17549)
Supplement: Supplementary file 1 — Data S1 [file APA-114-1222-s001.docx]

**Study Protocol**

**FULL/LONG TITLE OF THE STUDY**

Does closed-loop automated oxygen control reduce the duration of mechanical ventilation? A randomised controlled trial in ventilated preterm infants

**SHORT STUDY TITLE / ACRONYM**

Optimising ventilation in preterms with closed-loop oxygen control

**PROTOCOL VERSION NUMBER AND DATE**

**Version 2.0, 17^th^ February 2022**

**RESEARCH REFERENCE NUMBERS**

| **IRAS Number: 297749** |  |
| --- | --- |
| **SPONSORS Number:** |  |
| **FUNDERS Number: N/A** |  |

**SIGNATURE PAGE**

The undersigned confirm that the following protocol has been agreed and accepted and that the Chief Investigator agrees to conduct the study in compliance with the approved protocol and will adhere to the principles outlined in the Declaration of Helsinki, the Sponsor’s SOPs, and other regulatory requirement.

I agree to ensure that the confidential information contained in this document will not be used for any other purpose other than the evaluation or conduct of the investigation without the prior written consent of the Sponsor

I also confirm that I will make the findings of the study publicly available through publication or other dissemination tools without any unnecessary delay and that an honest accurate and transparent account of the study will be given; and that any discrepancies from the study as planned in this protocol will be explained.

| **For and on behalf of the Study Sponsor:** | | |
| --- | --- | --- |
| Signature:  .............................................................................................. |  | Date: ....../....../...... |
| Name (please print):  .............................................................................................. |  |  |
| Position: .............................................................................................. |  |  |
| **Chief Investigator:** | | |
| Signature: .............................................................................................. |  | Date: ....../....../...... |
| Name: (please print):  .............................................................................................. |  |  |

#

# LIST of CONTENTS

| **GENERAL INFORMATION** | **Page No.** |
| --- | --- |
| TITLE PAGE | i |
| RESEARCH REFERENCE NUMBERS | i |
| SIGNATURE PAGE | ii |
| LIST OF CONTENTS | iii |
| KEY STUDY CONTACTS | iv |
| STUDY SUMMARY | v |
| FUNDING | v |
| ROLE OF SPONSOR AND FUNDER | vi |
| ROLES & RESPONSIBILITIES OF STUDY STEERING GROUPS AND INDIVIDUALS | vi-vii |
| LIST OF ABBREVIATIONS | vii |
| STUDY FLOW CHART | viii |
| SECTION | |
| 1. BACKGROUND | 1-3 |
| 2. RATIONALE | 3 |
| 3. RESEARCH QUESTION/AIM(S) | 3-4 |
| 4. STUDY DESIGN/METHODS | 4-6 |
| 5. STUDY SETTING | 6 |
| 6. SAMPLE AND RECRUITMENT | 7-8 |
| 7. ADVERSE EVENTS AND INCIDENT REPORTING | 9-13 |
| 8. ETHICAL AND REGULATORY COMPLIANCE | 13-15 |
| 9. DISSEMINATION POLICY | 16 |
| 10. REFERENCES | 17-18 |
| 11. APPENDICES | 18 |

# KEY STUDY CONTACTS

| Chief Investigator | Professor Anne Greenough  Neonatal Intensive Care Unit  King’s College Hospital, SE5 9RS  02071887188  anne.greenough@kcl.ac.uk |
| --- | --- |
| Study Co-ordinator | Professor Anne Greenough  Neonatal Intensive Care Unit  King’s College Hospital, SE5 9RS  02071887188  anne.greenough@kcl.ac.uk |
| Sponsor | Professor Reza Razavi  Director of Research  Management & Director of Administration (Health Schools), Room 5.31, James Clerk Maxwell Building, 57 Waterloo Road, London SE1 8WA  Tel: +44 (0)2078483224  Email: [reza.razavi@kcl.ac.uk](mailto:reza.razavi@kcl.ac.uk)  PA email: susan.dickson@kcl.ac.uk |
| Joint-sponsor(s)/co-sponsor(s) | Rahman Ahmed  Research and Innovation Governance Manager  King’s College Hospital NHS Foundation Trust  161 Denmark Hill, London, SE5 8EF  e-mail: rahman.ahmed1@nhs.net |
| Funder(s) | N/A |
| Key Protocol Contributors | Dr Ourania Kaltsogianni, Neonatal Grid Trainee and research fellow in Neonatal Medicine, King’s College London  Dr Theodore Dassios, Consultant Neonatologist, King’s College Hospital, London |
| Committees | n/a |

**STUDY SUMMARY**

| **STUDY OVERVIEW** | |
| --- | --- |
| Study Title | Does closed-loop automated oxygen control reduce the duration of mechanical ventilation? A randomised controlled trial in ventilated preterm infants |
| Internal ref. no. (or short title) | Optimising ventilation in preterms with closed-loop oxygen control |
| Study Design | Randomised controlled trial |
| Study Participants | Preterm infants born at less than 31 weeks completed gestation requiring mechanical ventilation |
| Planned Size of Sample (if applicable) | 70 infants (2 groups) |
| Follow up duration (if applicable) | Participants will be followed up till their discharge from the neonatal unit |
| Planned Study Period | Two years |
| Research Question/Aim(s) | To identify if the use of automated closed loop oxygen saturation monitoring compared to standard care will reduce the duration of mechanical ventilation in preterm infants. |
| **DEVICE INFORMATION** | |
| Device Name | Oxygenie Auto-O_2_ software, Part No: Z6000/ CLP |
| Manufacturer Name | SLE Limited |
| Principal Intended Use | The 'Oxygenie' is a closed loop automated oxygen control system that has been incorporated into a software module for the SLE6000 infant ventilators. This software control system allows targeting SpO2 values by controlling FiO2. |
| Length of time the device has been in use | 3 years |

**FUNDING AND SUPPORT IN KIND**

| **FUNDER(S)**  (Names and contact details of ALL organisations providing funding and/or support in kind for this study) | **FINANCIAL AND NON FINANCIALSUPPORT GIVEN** |
| --- | --- |
| No external funding required |  |

**ROLE OF STUDY SPONSOR AND FUNDER**

King’s College London will take primary responsibility for ensuring the study design meets appropriate standards and uses proper conduct and reporting. King’s College London also provides insurance cover to provide for payment of damages or compensation in respect of any claim made by a research subject for bodily injury arising out of participation in a clinical trial or healthy volunteer study with certain restrictions.

King’s College Hospital takes responsibility for arranging the initiation and management of the research, and will ensure that appropriate standards, conduct and reporting are adhered to with regards to its facilities and staff involved with the study. King’s College Hospital will also undertake the governance review for the project and provide cover for clinical negligence by any of its staff undertaking the research.

**ROLES AND RESPONSIBILITIES OF STUDY MANAGEMENT COMMITEES/GROUPS & INDIVIDUALS**

There are no committees or steering groups involved in the management of this study at present.

**PROTOCOL CONTRIBUTORS**

This study has been designed by Professor Anne Greenough and Dr Theodore Dassios. Dr Ourania Kaltsogianni, neonatal grid trainee and research fellow, will conduct the study and primarily perform data analysis and interpretation, manuscript writing and dissemination of results with support from both supervisors.

| **KEY WORDS:** | Neonatology, closed-loop, oxygen, ventilation |
| --- | --- |

**LIST OF ABBREVIATIONS:**

| AE | Adverse event |
| --- | --- |
| ADE | Adverse device effect |
| BPD | Bronchopulmonary dysplasia |
| CI | Chief Investigator |
| CLAC | Closed-loop automated oxygen control |
| CPAP | Continuous positive airway pressure |
| CRF | Case report form |
| FiO_2_ | Fraction of inspired oxygen |
| KCL | King’s College London |
| NICU | Neonatal Intensive Care Unit |
| NIPPV | Non-invasive positive pressure ventilation |
| PI | Principal Investigator |
| REC | Research Ethics Committee |
| ROS | Reactive oxide species |
| SAE | Serious adverse event |
| SADE | Serious adverse device effects |
| SpO_2_ | Oxygen saturation |

#

# STUDY FLOW CHART

Ventilated infants< 31 weeks gestation admitted to NICU at King’s College Hospital

Resume ventilation with Oxygenie if < 28 days old

Randomisation with online computer generated random number

Ventilation with Oxygenie, adjusted by clinical staff as necessary

Screened as eligible by clinical team (no congenital abnormalities)

Parent/ legal guardian approached and consents to the study

Duration of mechanical ventilation

Oxygen saturation and FiO_2_ data from ventilator

Overall time spent on oxygen

Development of BPD

Length of stay

Successful extubation

Reintubation within 48 hours

Extubation to CPAP or NIPPV as per unit’s protocol

Standard ventilation adjusted manually as per unit’s protocol

# BACKGROUND

Seven percent of all infants are born prematurely and many require respiratory support in the newborn period. Unfortunately, although such support can be life- saving, premature infants who require mechanical ventilation frequently develop complications. The most common adverse outcome is bronchopulmonary dysplasia (BPD, oxygen dependency at 36 weeks post menstrual age) and importantly prematurely born infants can suffer chronic respiratory morbidity including troublesome respiratory symptoms, lung function abnormalities and exercise intolerance even in adolescence and adulthood [1]. Other complications include retinopathy of prematurity which can cause blindness and intracerebral haemorrhage which can result in cerebral palsy.

Oxygen saturation monitoring

Neonates with respiratory distress frequently require supplementary oxygen, but its use can result in the development of reactive oxide species (ROS) which increase the risk of complications such as bronchopulmonary dysplasia and retinopathy of prematurity. Targeting oxygen therapy to maintain oxygen saturations (SpO_2_) within a predefined range (SpO_2_ of 90-95%) can maximise the benefits of increased oxygen delivery to tissues whilst minimising the risk of complications. As a consequence, in clinical practice, peripheral oxygen saturations are continuously monitored and used to guide adjustments to the inspired oxygen (FiO_2_), which are made manually by neonatal practitioners. Neonatal patients are prone to frequent fluctuations with as many as 600 intermittent hypoxic episodes in one week documented in one study [2]. Compliance with SpO_2_ target in oxygen saturations ranges has been shown to be variable even within the same patient over time, as well as between patients and centres [3]. One study demonstrated that their target oxygen saturation achievement was as low as 20% [4]. Furthermore, target achievement decreases as the number of patients per nurse increases [5].

Automated closed loop oxygen control delivery

Closed loop automated oxygen control (CLAC) systems use SpO_2_ values monitored in real time, to calculate and make an adjustment to the (FiO_2_) without any human intervention. The resultant change in SpO_2_ is monitored and further alterations to the FiO_2_ made as needed. Closed loop automated oxygen control systems may, therefore, provide a solution for the low compliance to target oxygen levels, reduce the need for manual adjustments (and hence workload) and decrease complications. Indeed, in a randomised crossover study, we demonstrated that during the automated oxygen control period, there were fewer prolonged desaturations, infants spent a higher proportion of the time within their target SpO_2_ range during and fewer manual adjustments were made to the inspired oxygen concentration [6]. Our literature review identified 18 studies and highlighted that CLAC was associated with an increased percentage of time spent within the target oxygen saturation range, but there were no data to determine whether CLAC would reduce important clinical outcomes [7]. A previous review of 16 studies highlighted that the studies of CLAC were very heterogenous for design, population size and device used and also emphasised none had demonstrated whether the clinical outcome of preterm infants was improved. In our study, we demonstrated that the inspired oxygen concentration was reduced more rapidly than compared to manual control; the inspired oxygen concentration is a major determinant of when infants are considered to be ready for extubation [8]. We, therefore, now propose to undertake a randomised controlled trial to determine if the use of CLAC is associated with a reduction in the duration of mechanical ventilation. This is an important outcome as we have demonstrated that infants ventilated for more that seven days develop BPD [9].

# 2 RATIONALE

As above, previous studies demonstrated that closed-loop automated oxygen control has potential to help reduce episodes of severe prolonged desaturations and increases the time spent within the target SpO_2_ range with fewer manual adjustments required by clinical staff when compared to manually controlled ventilation. Further, we demonstrated that CLAC was associated with a more rapid reduction of the inspired oxygen concentration when compared to manual control. The inspired oxygen concentration is a major determinant of readiness for extubation. Hence, CLAC may be effective in reducing the duration of mechanical ventilation that is an important clinical outcome linked to long-term comorbidities. That would be a novel finding as previous studies have not reported on the effect of CLAC on clinical outcomes of preterm infants.

# RESEARCH QUESTION/AIM(S)

To explore if in prematurely born ventilated infants at a tertiary neonatal intensive care unit, the use of closed loop automated oxygen control compared to standard care reduces the duration of mechanical ventilation.

**3.1** **Objectives**

The primary objective of this study is to compare the duration of mechanical ventilation in preterm infants receiving closed-loop automated oxygen control with those receiving manually-adjusted oxygenation.

Secondary objectives are to evaluate any differences between the two groups in time spent in target oxygen saturation ranges, incidence of hypoxia or hyperoxia, manual adjustments required by clinical staff and adverse outcomes including BPD (at 36 weeks post menstrual age), overall duration of oxygen supplementation and length of stay.

- 1. **Outcome**
- Our primary outcome is the duration of mechanical ventilation
- Secondary outcomes are:

-percentage of time spent in target saturation range

-time spent in hypoxia and hyperoxia

-number of manual adjustments required

-number of days on oxygen

-diagnosis of BPD at 36 weeks post menstrual age

-length of ICU stay

# 4 STUDY DESIGN

This will be a randomised controlled trial. We aim to recruit a minimum of seventy premature ventilated infants born at less than 31 weeks gestation. Participants will be randomised to either closed-loop automated oxygen control or manually controlled oxygen from recruitment to successful extubation. We will also record basic epidemiologic parameters such as gestation at birth, birth weight, corrected gestational age, day of life and weight at study enrolment, use of antenatal steroids, mode of delivery, doses of surfactant administered, birth plurality and associated comorbidities that may impact on the duration of mechanical ventilation such as open ductus arteriosus and current evidence of infection.

Infants with known congenital anomalies will be excluded from the study.

Informed written consent will be requested from the parents or legal guardians of the infants and the attending Neonatal Consultant will be requested to assent to the study.

**METHODOLOGY**

When the clinical team identifies an infant is eligible for enrolment to the study and following verbal assent of the attending neonatal consultant, a member of clinical staff will initially approach the parents/ legal guardians of eligible infants and if they agree, a researcher. The parents will be provided with an information sheet about the study. The researchers will answer questions and respond to any concerns in a face-to-face meeting. Written informed consent will be obtained.

Eligible infants whose parents consent to the study will be enrolled within 48 hours of initiation of mechanical ventilation. Infants who have not been eligible for recruitment within 48 hours of initiation of mechanical ventilation (for example outborn infants transferred to our unit at a later date) but who remain ventilated on day seven of life and beyond, they will be enrolled to the study immediately after obtaining parental consent.

Randomisation will be performed using an online randomisation generator.

Patients will be ventilated using SLE6000 ventilators. Ventilation settings will be manually adjusted by the clinical team as per unit’s protocol. The intervention group, in addition to standard care will be also connected to the OxyGenie closed-loop oxygen saturation monitoring software (SLE). This software uses oxygen saturations from the SpO_2_ probe attached to the neonate, fed into an algorithm, to automatically adjust the percentage of inspired oxygen to maintain oxygen saturations within the target range. Manual adjustments including the percentage of FiO_2_ will be allowed at any point during the study if deemed appropriate by the clinical team.

The nurse-to-patient ratio will be according to the unit’s protocol that is determined on the patient’s acuity.

Patients will be studied from enrolment until successful extubation [10]. If an infant fails extubation and requires reintubation within 48 hours, he will be studied in his initial arm if less than 28 days old. Therefore, for those infants randomised at the intervention group CLAC will resume. Preterm infants that remain ventilated beyond day 28 of life will continue at their study arm (closed-loop automated oxygen control or manual oxygen control) till their first extubation attempt.

The infant’s clinical team will be present throughout the study to advise and trouble-shoot.

Continuous data will be compared between infants in the intervention group (closed-loop automated oxygen control) and infants in the control group (manual oxygen control). Student's t test will be used for normally distributed data and Mann-Whitney U test for skewed data. Categorical data will be assessed using the Fisher two tailed exact test. P values less that 0.05 will be considered statistically significant. Statistical analyses will be performed using SPSS software (SPSS Inc, Chicago, Illinois, USA).

Notes will be stored in a locked filling cabinet. All identifiable information will be stored in password protected computers and on encrypted devices.

# 5 STUDY SETTING

This is a single-centre study. Recruitment, intervention and data collection will take place on the Neonatal Intensive Care Unit at King’s College Hospital, Denmark Hill, London. The unit routinely cares from preterm ventilated infants and has previous experience on the use of OxyGenie in the context of trials. Participants will be identified by clinical staff and their parents will be approached and consented by the investigators.

**6 SAMPLE AND RECRUITMENT**

- 1. **Eligibility Criteria**

**6.1.1 Inclusion criteria**

- Preterm infants less than 31 weeks completed gestation at birth requiring mechanical ventilation and admitted to King’s NICU in the first 48 hours after birth
- Preterm infants less than 31 weeks completed gestation at birth requiring mechanical ventilation on day seven of life and beyond and who have not been eligible for recruitment to the study in the first 48 hours after birth

**6.1.2 Exclusion criteria**

- Preterm infants above 31 weeks completed gestation or term born infants
- Infants with major congenital abnormalities

**6.2 Sampling**

**6.2.1 Size of sample**

In a cohort of 368 preterm infants born between 24 to 30 weeks gestation, the standard deviation for the duration of mechanical ventilation was 3.83 days [11]. To detect a decrease in the duration of mechanical ventilation of three days with a power of 90% and a significance level of 5%, a sample size of 35 infants in each group would be required.

Historical data from our unit for the period 2015-2020 demonstrated that the median duration of mechanical ventilation for preterm infants born less than 31 weeks gestation was 12 days. Based on the unit’s clinical activity in the most recent years, more than 100 preterm infants less than 31 weeks completed gestation were admitted on average at King’s College Hospital over a twelve month period. An interim analysis will be carried out at half sample size to review if enough data have been gained at that point. The analysis will be performed by an independent researcher not involved in the project to avoid introducing bias to our data. The research team will be blinded to the results of the interim analysis unless there are any significant findings.

**6.2.2 Sampling technique**

All eligible patients will be approached and included in the study following parents/legal guardians’ consent and assent of the attending Neonatal Consultant.

Parents of eligible patients will be approached and consented within 24 hours of initiation of mechanical ventilation or as soon as an infant is screened as eligible for the study on day seven of life and beyond. If preterm delivery is anticipated, parents may be approached antenatally and consented once the infant is mechanically ventilated at birth.

**6.3 Recruitment**

**6.3.1 Sample identification**

The clinical team will identify potential participants and alert the research team. The latter will not access any patient identifiable information or medical notes until parents/ legal guardians have consented.

No payment will be made to participants or their families as the study will not require any additional visits/ inconvenience.

**6.3.2 Consent**

- The nature, objectives and process of the study, including possible risks, will be discussed by the chief investigator with patient’s parents/ carers
- An information leaflet will be provided to the parents/ carers detailing the above in addition to the consent documents
- Parents/ carers will be encouraged to ask questions throughout the consenting process and study, including after the study period has finished

1. **ADVERSE EVENTS AND INCIDENT REPORTING**

**7.1 Definition of Adverse Events**

| **Term** | **Definition** |
| --- | --- |
| Adverse Event (AE) | Any untoward medical occurrence in a patient or study participant, which does not necessarily have a causal relationship with the intervention/treatment/procedure involved. |
| Adverse Device Effect (ADE) | All untoward and unintended responses to a medical device.  The phrase "responses to a medical device" means that a causal relationship between the device under investigation and an AE is at least a reasonable possibility, i.e., the relationship cannot be ruled out.  All cases judged by either the reporting medically qualified professional or the sponsor as having a reasonable suspected causal relationship to the device qualifies as a device effect.  This also includes any event resulting from insufficiencies or inadequacies in the instruction for use or deployment of the device and includes any event that is a result of a user error. |
| Serious Adverse Event (SAE). | Any adverse event that:   - results in death, - is life-threatening*, - requires hospitalisation or prolongation of existing hospitalisation**, - results in persistent or significant disability or incapacity, or - consists of a congenital anomaly or birth defect |
| *A life- threatening event, this refers to an event in which the participant was at risk of death at the time of the event; it does not refer to an event which hypothetically might have caused death if it were more severe.  ** Hospitalisation is defined as an in-patient admission, regardless of length of stay. Hospitalisation for pre-existing conditions, including elective procedures do not constitute an SAE. | |
| Serious Adverse Device Effects (SADE): | A serious adverse device effect (SADE) is any untoward medical occurrence seen in a patient that can be attributed wholly or partly to the device, which resulted in any of the characteristics or led to characteristics of a Serious adverse event.  SADE is also any event that may have led to these consequences if suitable action had not been taken or intervention had not been made or if circumstances has been less opportune. All cases are to be assessed by either the reporting medically qualified professional or the sponsor. |

## **7.2 Assessment and management of risk**

Potential risks include:

- Failure of the closed-loop monitoring software: that would result in the patient’s ventilation changed to manual control and it should not impact the quality of clinical management. It would be recorded as an adverse event.
- Failure of data recording by the ventilator. We will minimise the risk by testing each ventilator’s data recording process prior to the commencement of the monitoring period.
- Disclosure of a safeguarding risk: any such information will be disclosed to the unit’s Safeguarding Lead and the clinical team caring for the patient and managed as per local policy.
- Closed-loop automated oxygen control could potentially mask an increased oxygen requirement between manual observation recordings as oxygen saturation levels remain within their target range. The system we will use will activate an alarm if there is an increase in FiO_2_≥30% from the basal level.

**7.3 Recording and reporting of adverse events and serious adverse events**

All adverse events will be recorded in the medical records in the first instance and then in the case report form (CRF). All adverse events will be recorded with a simple, brief description of the event, including dates as appropriate and any clinical symptoms observed.

All serious adverse events will be recorded on a serious adverse event (SAE) form. The CI/PI or designated individual will complete the sponsor’s SAE form and the form will be preferably emailed to the sponsor within one working day of becoming aware of the event. The CI/PI will respond to any SAE queries raised by the sponsor as soon as possible. When the event is unexpected and thought to be related to the use of the device, this must be reported by the CI/ Sponsor to the ethics and HRA within fifteen days.

**7.4 Reporting urgent safety measures**

If any urgent safety measures are taken the CI shall immediately and in any event no later than three days from the date the measures are taken, give written notice to the relevant REC and sponsor of the measures taken and the circumstances giving rise to those measures.

**7.5 Protocol deviations and notification of reportable protocol violations**

The CI will monitor protocol deviations. A protocol violation is a breech that is likely to affect to a significant degree:

1. the safety or physical or mental integrity of the participants; or
2. the scientific value of the study.

The sponsor will be notified immediately of any case where the above definition applies during the study conduct phase.

**7.6 Trust incidents and near misses**

An incident or near miss is any unintended or unexpected event that could have or did lead to harm, loss or damage that contains one or more of the following components:

a. It is an accident or other incident which results in injury or ill health.

b. It is contrary to specified or expected standard of patient care or service.

c. It places patients, staff members, visitors, contractors or members of the public at unnecessary risk.

d. It puts the Trust in an adverse position with potential loss of reputation.

e. It puts Trust property or assets in an adverse position or at risk.

Incidents and near misses must be reported to the Trust through DATIX as soon as the individual becomes aware of them.

A reportable incident is any unintended or unexpected event that could have or did lead to harm, loss or damage that contains one or more of the following components:

1. It is an accident or other incident which results in injury or ill health.
2. It is contrary to specified or expected standard of patient care or service.
3. It places patients, staff members, visitors, contractors or members of the public at unnecessary risk.
4. It puts the Trust in an adverse position with potential loss of reputation.
5. It puts Trust property or assets in an adverse position or at risk of loss or damage.

# 8 ETHICAL AND REGULATORY CONSIDERATIONS

We anticipate that ethical concerns will not arise in the context of this study. All participants will receive standard care in terms of ventilation equipment and monitoring by clinical staff. Ventilator settings will be adjusted and other interventions such as blood gases or chest Xrays will be performed as per clinician’s discretion. Therefore, there should not be any risk imposed to the intervention group with the addition of closed-loop monitoring. In addition, data collection will not be intrusive to patients as data will be stored in and exported from the ventilator software and no extra observations will be required. Data collected from medical notes will be anonymised.

**8.1 Expected outcomes of the study**

The potential benefit to the patient is that closed-loop automated oxygen control will allow earlier weaning of the inspired oxygen concentration provided, earlier extubation and potentially reduced risk of comorbities related to prolonged ventilation. As we demonstrated in a previous randomised crossover study, patients on closed-loop monitoring of oxygenation also benefited from increased time spent within their target oxygen saturation zone, fewer prolonged episodes of desaturations and required less manual interventions from nursing staff that could allow them to carry out other duties.

**8.2 Research Ethics Committee (REC) and other Regulatory review & reports**

Before the start of the study, a favourable opinion will be sought from a REC for the study protocol, and other relevant documents (informed consent forms and patient information leaflets).

**8.3 Regulatory Review & Compliance**

The Chief Investigator will ensure that appropriate approvals from participating organisations are in place before commencing the study.

For any amendment to the study, the Chief Investigator or designee, in agreement with the sponsor will submit information to the appropriate body in order for them to issue approval for the amendment.

**8.4 Peer review**

This study has been reviewed within the institution’s clinical research team.

**8.5 Patient & Public Involvement**

We have involved parents/ carers in NICU to comment on the study design during its development.

**8.6 Protocol compliance**

Any accidental protocol deviations will be adequately documented and reported to the Chief Investigator and Sponsor immediately. The Chief Investigator and sponsor will monitor and audit the conduct of this research.

**8.7 Data protection and patient confidentiality**

The General Data Protection Regulation and Data Protection Act 2018 will be adhered to. Data will be de-identified before being entered in a secure database. A unique study identifier number will be issued to each participant on enrolment into the study. That number will be used in all subsequent data collection forms. Patient paper data will be stored in a locked filing cabinet in a room that is only accessible to the research team and that is based at the Neonatal Intensive Care Unit facilities at King's College Hospital. Patient de-identified electronic data will be stored on encrypted university computers or memory stick devices, both of which require user identification and password verification.

**8.8 Archiving**

At the end of the trial, all essential documentation will be archived securely by the CI for a minimum of 25 years from the declaration of the end of the trial. Essential documents include those which enable both the conduct of the trial and the quality of the data produced to be evaluated and show whether the site complied with all applicable regulatory requirements. All archived documents will continue to be available for inspection by appropriate authorities upon request.

8.9 Indemnity

King’s College London indemnity applies for insurance/ indemnity to meet the potential legal liability of the sponsor for harm to participants arising from the design and management of the research. NHS indemnity scheme applies for insurance/ indemnity to meet the potential legal liability of the investigators arising from harm to participants in the conduct of the research.

**8.10 Access to the final study dataset**

The individuals in the study will be notified of the outcome of the study as below. The investigators will have access to the final study dataset. Participants will be informed that anonymised data may be shared with other researchers for research purposes only.

### 9 DISSEMINIATION POLICY

### 9.1 Dissemination policy

On completion of the study, the data will be analysed and a final study report will be prepared that could be accessed via the sponsor. The study will be presented at research meetings at the Neonatal Intensive Care Unit at King’s College Hospital as well as university meetings at King’s College London. Anonymised study data may be presented at conferences and published by the investigators at peer reviewed journals. The results will also be disseminated in the form of the MD dissertation of Dr Kaltsogianni. Participants will be notified of the outcome of the study via provision of the publication and an accompanying newsletter.

**9.2 Authorship eligibility guidelines and any intended use of professional writers**

### The final study authors will include Dr Ourania Kaltsogianni, Dr Theodore Dassios and Professor Anne Greenough.

**9.3 Intellectual property**

All intellectual property rights and know-how in the protocol and in the results arising directly from the study shall belong to KCL.

### 10 REFERENCES

1. Greenough A. The long-term respiratory consequences of very premature birth (<32 weeks gestation). Early Hum Dev 2013;89:S25-7.
2. Di Fiore JM, Bloom JN, Orge F, Schutt A, Schluchter M, Cheruvu VK, et al. A higher incidence of intermittent hypoxemic episodes is associated with severe retinopathy of prematurity. J Pediatr 2010;157:69–73.
3. Hagadorn JI, Furey AM, Nghiem T-H, Schmid CH, Phelps DL, Pillers D-AM, et al. Achieved versus intended pulse oximeter saturation in infants born less than 28 weeks’ gestation: The AVIOx study. Pediatrics 2006;118:1574–82.
4. Ford SP, Leick-Rude MK, Meinert KA, Anderson B, Sheehan MB, Haney BM, et al. Overcoming barriers to oxygen saturation targeting. Pediatrics 2006;118:S177–86.
5. Sink DW, Hope SAE, Hagadorn JI. Nurse: Patient ratio and achievement of oxygen saturation goals in premature infants. Arch Dis Child Fetal Neonatal Ed 2011;96:96–101.
6. Sturrock S, Ambulkar H, Williams E, Sweeney S, Bednarczuk N, Dassios T, Greenough A. A randomised crossover trial of closed loop automated oxygen control in preterm, ventilated infants. Acta Paediatr 2020 (in press).
7. Sturrock S, Williams E, Dassios T, Greenough A. Closed loop automated oxygen control in neonates – a review. Acta Paediatr 2020;109:914-22.
8. Dani C. Automated control of inspired oxygen (FiO_2_) in preterm infants – a literature review. Pediatr Pulmonol 2019;54:358-63.
9. Hunt K, Dassios T, Ali K, Greenough A. Prediction of bronchopulmonary dysplasia development. Arch Dis Child Fetal Neonatal Ed 2018;103:F598-9.
10. Dimitriou G, Greenough A, Endo A, Cherian S, Rafferty GF. Prediction of extubation failure in preterm infants. Arch Dis Child Fetal Neonatal Ed 2002; 86: F32-35.
11. Vliegenthart RJS, van Kaam AH, Aarnoudse-Moens CSH, van Wassenaer AG, Onland W. Duration of mechanical ventilation and neurodevelopment in preterm infants. Arch Dis Child Fetal Neonatal Ed 2019; 104: F631-F635.

**11. APPENDICES**

**10.1 Appendix 1- Required documentation**

- Patient information sheet
- Consent form
  1. **Appendix 2 – Schedule of Procedures (Example)**

| **Procedures** |  |  |  |
| --- | --- | --- | --- |
|  | **Screening** | **Baseline** | **Time of discharge to home** |
| Informed consent | x |  |  |
| Demographics |  | x |  |
| Medical history |  | x |  |
| Observation of treatment |  | x |  |
| Review of outcome |  |  | x |

**10.3** **Appendix 3 – Amendment History**

| Amendment No. | Protocol version no. | Date issued | Author(s) of changes | Details of changes made |
| --- | --- | --- | --- | --- |
|  |  |  |  |  |
